# Predicting life expectancy after geriatric hip fracture: A systematic review

**DOI:** 10.1371/journal.pone.0261279

**Published:** 2021-12-15

**Authors:** Alexander Lee, Sara Weintraub, Ianto Lin Xi, Jaimo Ahn, Joseph Bernstein

**Affiliations:** 1 Department of Orthopaedic Surgery, University of Pennsylvania, Philadelphia, Pennsylvania, United States of America; 2 Corporal Michael J. Crescenz VAMC, Philadelphia, Pennsylvania, United States of America; University Hospital Leipzig, GERMANY

## Abstract

**Background:**

Displaced femoral neck fractures in geriatric patients are typically treated with either hemiarthroplasty or total hip arthroplasty. The choice between hemiarthroplasty and total hip arthroplasty requires a good estimate of the patient’s life expectancy, as the recent HEALTH trial suggests that the benefits of the two operations do not diverge, if at all, until the second year post-operatively. A systematic review was this performed to determine if there sufficient information in the medical literature to estimate a patient’s life expectancy beyond two years and to identify those patient variables affecting survival of that duration.

**Methods:**

Pubmed, Embase, and Cochrane databases were queried for articles reporting survival data for at least two years post-operatively for at least 100 patients, age 65 or greater, treated surgically for an isolated hip fracture. A final set of 43 papers was created. The methods section of all selected papers was then reviewed to determine which variables were collected in the studies and the results section was reviewed to note whether an effect was reported for all collected variables.

**Results:**

There were 43 eligible studies with 25 unique variables identified. Only age, gender, comorbidities, the presence of dementia and fracture type were collected in a majority of studies, and within that, only age and gender were reported in a majority of the results. Most (15/ 25) variables were reported in 5 or fewer of the studies.

**Discussion:**

There are important deficiencies in the literature precluding the evidence-based estimation of 2 year life expectancy. Because the ostensible advantages of total hip arthroplasty are reaped only by those who survive two years or more, there is a need for additional data collection, analysis and reporting regarding survival after geriatric hip fracture.

## Introduction

Geriatric hip fracture is a common and clinically significant problem worldwide [1]. These fractures can be broadly divided into two classes: intertrochanteric fractures and fractures of the femoral neck. Intertrochanteric fractures and non-displaced femoral neck fractures are usually treated with some form of fixation, and displaced femoral neck fracture with some form of joint replacement. Among patients with displaced femoral neck fractures, the surgical options include replacing the femoral head with a prosthesis, hemiarthroplasty, or replacing both the femoral head and the acetabulum with prostheses, namely, total hip arthroplasty.

The relative advantages of hemiarthroplasty and total hip arthroplasty, as revealed by systematic reviews and a meta-analyses of randomized controlled trials [2–4], can be summarized as follows: total hip arthroplasty surgery is a more complicated and costly procedure with a greater risk of post-operative dislocation. On the other hand, total hip arthroplasty offers better long term function and longevity.

If that rule holds, the apt choice between hemiarthroplasty and total hip arthroplasty requires a good estimate of the patient’s life expectancy. After all, if the benefits of total hip replacement are reaped only in the long run, hemiarthroplasty is the clearly preferred operation among patients with a shorter life expectancy.

A recent prospective controlled trial, the HEALTH study [5], suggests that the two-year life expectancy is an important line of demarcation. In this study, 1,495 patients with a displaced femoral neck fracture were randomized to undergo either total hip arthroplasty or hemiarthroplasty. The rate of secondary hip procedures–the main study outcome—was similar (about 8%) in both groups within two years of follow-up. As such, segregating patients on the basis of 2-year life expectancy is a critical clinical task.

The main question addressed in this study, accordingly, is whether the medical literature can reasonably inform an accurate estimate of a patient’s life expectancy beyond two years. Our secondary aim was to identify which variables were found to affect survival of that duration. To address these questions, we conducted a systematic review examining studies reporting survival for two or more years following surgical intervention for geriatric hip fracture.

## Methods

We assembled the set of papers describing patient survival following surgical intervention for hip fracture two years or more after injury among patients 65 years of age and older. This review was pre-registered with PROSPERO (CRD42020199661).

### Search strategy

We employed a comprehensive literature search of the Pubmed, Embase, and Cochrane databases for all articles published between 1/1/2000 and 7/20/20. The Cochrane databases were searched for the term “[Hip Fractures] explode all trees and with qualifiers(s): [mortality—MO]”; the search strategies used for Pubmed and Embase are shown in Tables 1 and 2. Our initial search yielded 2741 distinct records.

**Table 1:** Search strategy Pubmed.

1	Aged [MeSH Terms]
2	Aged, 80 and over [MeSH Terms]
3	Hip Fractures [MeSH Terms]
4	Mortality [MeSH Terms]
5	Survival analysis [MeSH Terms]
6	Hip Fractures/ Mortality[MeSH Terms]
7	Arthroplasty, Replacement, Hip/mortality[MeSH Terms]
8	1 or 2
9	4 or 5
10	3 and 9
11	10 or 6 or 7
**12**	**8 and 11**

**Table 2:** Search strategy Embase.

1	‘hip fracture’/exp
2	‘mortality’/exp
3	‘survival’/exp
4	‘death’/exp
5	‘very elderly’/exp
6	2 or 3 or 4
**7**	**1 and 6 and 5**

One of the authors then reviewed the abstracts of these 2,741 records to identify primary studies written in English that detailed a surgical intervention on at least 100 patients (an arbitrarily chosen criterion) and reported survival data for at least two years post-operatively. This review excluded 2530 records, yielding a set of 211 papers for further scrutiny.

**Fig 1:**
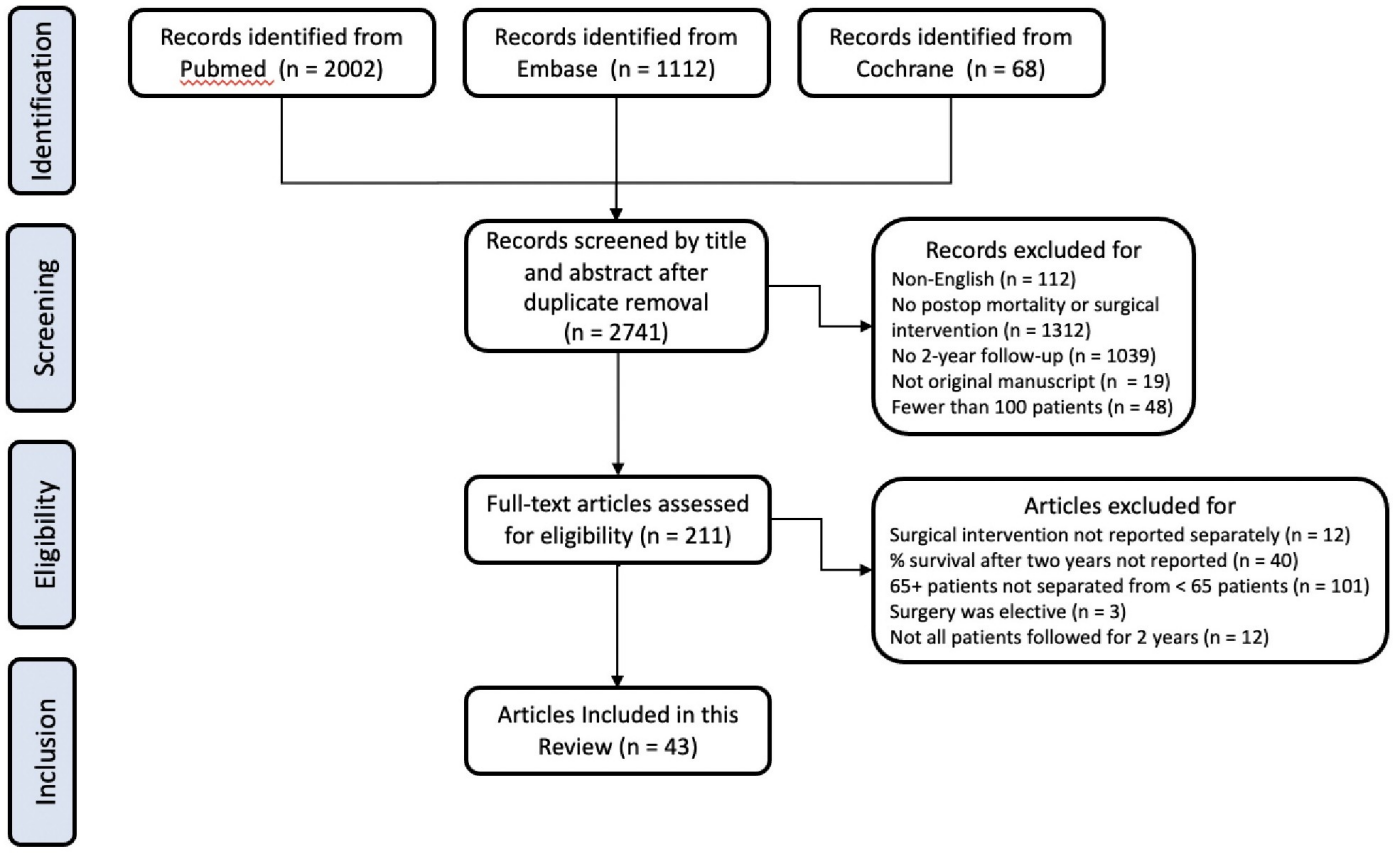
Flowchart of manuscript review.

The full-text manuscripts of these 211 papers were then reviewed to determine appropriateness for inclusion. From that full text review, we identified a final set of 43 papers that reported survival for at least two years following surgical intervention for isolated hip fracture among patients 65 years of age or more (Fig 1).

### Data extraction

The methods section of all selected papers were then reviewed by two authors to determine which variables were collected in the studies. From this review, a master list was created that included all pre-operative patient variables collected by at least one study in the set.

All manuscripts were then reviewed in parallel by two other reviewers. For each study, these reviewers noted whether a given variable on the master list was denoted in the Methods section to have been collected, and, if so, whether this variable was reported in the Results section. (For example, Alarcón et al [6] reported that 40.9% of fractures were intracapsular, 52.1% of fractures were intertrochanteric, and 7% were subtrochanteric, but did not report the influence of these fracture types upon post-operative survival. This study was therefore denoted to have “collected” the fracture-type variable but not having “reported” an effect of it.) Discrepancies were then resolved by an independent review of yet another reviewer.

These reviewers also extracted the country of origin, number of patients in all eligible patient cohorts, and minimum duration of follow-up from each study. From this, we were able to determine the number of manuscripts that reported survival of two or more years after hip fracture as well as the frequency with which each variable was collected and reported to influence survival.

Because the studies were assessed only for the presence or absence of reported variables—and not the values of these variables—there was no assessment of bias risk, effect measures, synthesis methods, reporting bias, or certainty.

## Results

There were 43 studies that reported survival rates for at least two years after surgical intervention for hip fracture in patients age 65 or older. Twelve of these studies reported survival rates for at least five years for all patients. There were 20 studies that limited their analysis to patients in a subset of the greater-than-65 patient population, eg, octagenarians [7], nonagenarians [8, 9], or centenarians [10]. Among the 43 studies, 26 were conducted on populations in Europe, seven in the United States, eight in east Asia, one in Israel [11], and one in New Zealand [12]. The combined population of the 43 studies was approximately 200,000 patients.

There were 25 unique variables identified on the master list (Table 3). All 43 studies collected at least one variable and 36 reported the predictive effect of at least one preoperative variable on postoperative survival. Only age, gender, comorbidities, the presence of dementia and fracture type were collected in a majority of studies, and within that, only age and gender were reported in a majority of the results.

**Table 3:** Master variable list.

Variable	“Collected”	“Reported”
	Number of studies that collected data for this variable (representing the number of patients shown in parentheses)	Number of studies that reported an effect of this variable on mortality (number of patients in these studies shown in parentheses)
Age	43	32
	(197963)	(189971)
Sex/gender	41	24
	(196668)	(117045)
Medical History: Any	30	18
	(178847)	(90782)
Medical History: Number, name of comorbidities	29	16
	(168963)	(79391)
Fracture pattern or type	22	12
	(75454)	(36179)
Dementia or cognitive decline	25	11
	(128193)	(36162)
Medical History: Charlson index	17	10
	(133055)	(55517)
ASA classification	16	9
	(8173)	(5049)
Hip function scores	19	6
	(6460)	(1736)
Living arrangements	10	5
	(33024)	(31743)
Number, name of medications	8	5
	(33413)	(32601)
Performance of activities of daily living	12	4
	(4662)	(1383)
Marital status	5	4
	(3038)	(2851)
Frailty Score	4	2
	(1299)	(467)
Weight/BMI	9	3
	(36741)	(31411)
Lab test results	9	2
	(3826)	(665)
Nutritional status	5	3
	(31583)	(31017)
Ethnicity	4	2
	(72191)	(1570)
Medical History: prior fracture	6	2
	(41424)	(30803)
Social history (eg smoking)	4	2
	(6942)	(2397)
Sarcopenia on imaging studies	1	1
	(187)	(187)
Social support	1	1
	(674)	(674)
Medical History: Osteoporosis	3	1
	(32358)	(30522)
Physiologic measurements (eg blood pressure)	2	1
	(829)	(450)

Notably, among those variables collected in more than 5 studies, the average rate of reporting was 48%. For example, information regarding fracture pattern or type was collected in 22 studies, yet only 12 reported results based on that information. The effect of patient age, a variable collected in every study, was reported in only 32 (74%).

There were nine variables (marital status, nutritional status, frailty score, ethnicity, social history, history of osteoporosis, physiologic measurements, sarcopenia and social support) that were recorded in five or fewer studies.

There were 32 studies that assessed the effect of factors that would be known only after the treatment decision has been made, for example, the presence of post-operative complications.

## Discussion

We assessed the literature available to help the clinicians estimate potential for survival beyond two years after geriatric hip fracture. We found that there were only 43 studies that reported 2 year survival data. To put this in perspective, there were 1,051 studies that were excluded from our initial search because the follow-up period was shorter than 2 years. We conclude that the medical literature offers relatively little guidance for the identification of patients apt to survive two years or more. Along those lines, although there are published data-derived decision rules to predict survival beyond two years for other musculoskeletal conditions (chondrosarcoma [13] for example), we were unable to identify a published decision rule for geriatric hip fracture survival at two years after injury.

Our secondary aim was to identify the frequency with which the variables thought to affect survival were reported. Here, we found that only two variables, age and sex, were reported in a majority of studies. By contrast, the remaining variables on our master list were reported in only a small minority of studies. Indeed, 15 of the 25 variables on the master list were reported in 5 or fewer of the studies in the set. If these variables in fact influence life expectancy, then complete collection and reporting of these data (even if no effect is seen in a particular study) will be helpful. More complete data reporting will promote more robust meta-analyses; it will also allow machine-learning methods to examine interactive effects that are not readily apparent in a single study [14].

Further, many variables that were collected by researchers were not included in their reported results. One fair inference is that the researchers did not find an effect and thus simply omitted further mention: an author-directed form of “positive-outcome bias” [15] within a published study. A requirement for providing all data (eg, as an appendix to a published report) might therefore improve secondary analyses.

As noted, 32 studies assessed the effect of factors that would be known only after the treatment decision has been made, such as the presence of post-operative complications [16]. While informative overall, factors discovered post-operatively cannot be used to make decisions pre-operatively.

The lack of studies addressing survival beyond two years is not an indictment of researchers and their priorities: until the HEALTH trial appeared, it may have been reasonably assumed that the differences between total hip arthroplasty and hemiarthroplasty emerge at the one-year point, for example, and there are many studies that can help identify those patients likely to live at least that long. Moreover, the published literature of course does offer help modulating one’s estimate of a given patient’s life expectancy. Papers that report, for example, how life expectancy is affected by sex, age, health index scores and indeed the presence of the fracture itself [17–20] are useful adjuncts to clinical estimates. These papers, nonetheless, do not on their own offer a life expectancy estimate for a given patient.

### Limitations

This study has limitations we have identified and likely others we have not. To start, the studies collected were not screened for quality. To a great extent, “quality” a subjective: a feature may be strength or a limitation, depending on context. For instance, if a study is highly focused on one population (e.g, Scandinavian males [21]) that may be an asset if the patient at hand is a member of that focused group, whereas in terms of its applicability to patients in general, that narrow focus is a liability. Because results of the studies were not used as a basis for inference here, the absence of a quality assessment is perhaps less critical, but it is still a limitation.

As with any systematic review that searches on a limited number of databases, it is certainly possible that studies may have been missed, especially those not in the English language.

Another putative limitation is that this systematic review reported on patients with both femoral neck and intertrochanteric fractures, whereas the question of life expectancy is germane to preoperative planning only for femoral neck fractures. One large study [22] investigated 6-month mortality among patients with femoral neck and intertrochanteric fractures and found that fracture type was not an independent predictor of mortality. Still, to the extent that this issue is relevant and that studies reporting on intertrochanteric fractures as well must be excluded, the larger conclusion about the inadequacy of the literature is buttressed: if papers not reporting results segregated by fracture are excluded, the set of studies remaining is that much smaller.

## Conclusion

Choosing the correct surgical treatment for a geriatric hip fracture of the femoral neck can be easy at times. For relatively frail or low-demand patients, hemiarthroplasty is likely to be the best choice. By the same token, when patients are healthy and their hips are arthritic, total hip replacement should be strongly considered. Between those extremes, though, lie the more difficult cases in which the patient’s life expectancy is a key consideration. That is because the ostensible advantages of total hip arthroplasty are reaped only by those who survive long enough. Emerging information suggests that defining life expectancies beyond two years is the critical task, as total hip arthroplasty and hemiarthroplasty might be sufficiently similar before that point. Nevertheless, the systematic review presented here suggests that the medical literature is insufficiently robust to guide evidence based practice. Moreover, it is likely that potentially-useful information has been collected but not reported. Taken together, the findings here suggest that additional data collection, analysis and reporting regarding medium-term survival after geriatric hip fracture, for points beyond two years, is needed.

## Supporting information

S1 File Study data. (XLSX)

S1 Checklist PRISMA checklist. (DOCX)
